# General Medicine and Therapeutics

**Published:** 1894-09

**Authors:** 


### *SELECTIONS AND ABSTRACTS.*

---

### GENERAL MEDICINE AND THERAPEUTICS.

Edited by Dr. Luther B. Grandy

### The Physiological Actions of Alcohol.

Dr. David Cerna, of the University of Texas, in an able paper on the physiological action of alcohol, read at the late Pan-American Congress, concluded as follows:

1. Alcohol in small amounts excites and in large doses depresses both the peripheral motor and sensory nerves.

2. Excessive quantities cause a spiral degeneration of the axis-cylinder of nerve-fibers.

3. Reflex action is at first increased and afterwards diminished by an influence exercised by the drug upon the spinal cord and the nerves.

4. In small amounts the drug stimulates the cerebral functions; it afterwards, especially in large quantities, depresses and finally abolishes them.

5. Alcohol causes lack of co-ordination by depressing both the brain and the spinal cord.

6. In toxic doses alcohol produces hyperemia of both brain and spinal cord, especially of the lumbar enlargement of the latter.

7. Small doses of alcohol produce increased rapidity of the cardiac beat; large amounts, a depression of the same. In either case the effect is brought about mainly through direct cardiac action.

8. The drug in small quantities cause a rise of the arterial pressure by a direct action upon the heart; in large amounts it depresses the arterial pressure similarly through a cardiac influence.

9. In large doses alcohol enhances coagulation of the blood; in toxic quantities it destroys the ozonizing power of this fluid, causing a separation of the hemoglobin from the corpuscles.

10. Alcohol in small doses has little or no effect on the respiratory function ; in large amounts it produces a depression of both rate and depth of the respiration through a direct action on the centers in the medulla oblongata.

11. The drug kills by failure of the respiration.

12. On the elimination of carbon dioxide alcohol exercises a varying action, sometimes increasing, sometimes decreasing, such elimination.

13. The action of alcohol on the amount of oxygen absorbed also varies, and may be said to be practically unknown.

14. The drug lessens the excretion of tissue waste, both in health and disease.

15. In small amounts alcohol increases the bodily temperature; in large doses it diminishes the same. The fall of the bodily temperature is due mainly to an excess of heat dissipation caused by the drug.

16. Alcohol, in sufficiently large amounts, has a decided antipyretic action.

17. In moderate amounts alcohol aids the digestive processes.

18. Alcohol diminishes the absorption of fats.

19. The drug exercises a varying influence on the amount of urine secreted, but it probably increases the activity of the kidneys.

20. In large doses, or when continuously used for a long time, alcohol produces cirrhotic changes of hepatic especially and paralysis of spinal origin. It also causes insanity, epilepsy, and other maladies.

21. Alcohol is mainly burnt up in the system when taken in moderate quantities, but when ingested in excessive amounts it is partly eliminated by the breath, the kidneys, and the intestines.

22. Alcohol is a conservator of tissue, a generator of vital force, and may therefore be considered as a food.

---

### THE BEST REMEDIES IN PHTHISIS.

Dr. C. W. Murphy contributes a good article to the *Louisville Medical Journal* (January), with the above title : A most useful remedy in phthisis, and one that it not used nearly so often as it should be, is sulphate of strychnine. I use 1-20 grain tablets by ordering a half of one (1-40) grain half an hour before meals for three days, then a whole one for a like period and many cases can bear one and a half tablets at a dose without experiencing the physiological effects. It should be long continued. It is an appetizer, promotes good digestion and is beyond question a blood and tissue builder. After meals I give maltine and cod liver oil in tablespoonful doses, and if the stomach should be very weak, hydroline instead. All preparations containing oil should be administered an hour or an hour and a half after meals, as the oil is digested in the intestinal canal and not in the stomach; and if given immediately after eating it often seriously interferes with the digestion of albuminoids. Give a tablespoonful or more of apple brandy or whisky saturated with rock candy, just afterwards, for it serves the triple purpose of promoting the acceptability of the oil by the stomach, aiding its digestion and ameliorating the cough, to say nothing of its action as a stimulant. With the maltine and oil, or hydroline, I often mix with each dose from one to eight drops of beechwood creosote. This remedy seems to be signally beneficial in some cases, while in others the results are *nil.* Compound syrup of the hypophosphites is an old-time medicine, to be used before or after meals. For night sweats I have never found a better remedy than $\frac{1}{8}$ of a grain of agaricin, three times a day. Atropine sulphate in 1-125 to 1-100 of a grain at bedtime is excellent but for the disagreeable dryness of the mouth and throat which it generally produces. Another reliable remedy is camphoric acid in twenty grain doses two hours before the expected sweating. Many other remedies might be mentioned which are useful for special symptoms, as phenacetine in eight or ten grain doses to moderate the fever, palliate nervous symptoms and make the patient generally more comfortable; quinine in decided doses, two or three, for a few days, given with reference to the paroxysm, will often prevent chills and lessen the evening temperature. The importance of being much of the time in the open air, mild exercises short of fatigue, bathing followed by friction, counter-irritation over painful pulmonary areas; nutritious, easily digested food in addition to measures already suggested, need only be mentioned. As to climate, a dry, elevated, equable temperature is the place for consumptives.

### TREATMENT OF TONSILLITIS.

In the *Therapeutic Gazette* Dr. Slagle gives the following as his usual treatment of follicular tonsillitis.

If seen *very early* and no complications, Dr. Sajous' "*abortive* treatment" with ammoniated tincture of guaiac (a teaspoonful every two hours in sweet milk) will often (not always) be found quite satisfactory. If, however, called to see the patient later, which is generally the case, give

| | | |
|---|---|---|
| ℞ | Calomel | gr. x. |
| | Soda bicarb | gr. xx. |

M. ft. chart. No. iij. Sig.—One every three hours, floating on a teaspoonful of water, and no liquid afterward for twenty minutes.

These powders to be followed by a teaspoonful or two of castor oil with ten to fifteen drops of turpentine every hour (for first twelve hours), excepting the hour of the powders. Gargle and swallow a teaspoonful of a saturated solution of c. p. sulphate of sodium. After the powders are all used and worked off with the castor oil and the turpentine, alternate the solution of sulphite of sodium with the following chloride syrup, both as gargle and systemic remedy:

| | | |
|---|---|---|
| ℞ | Potass. chlorat | ʒ j. |
| | Ammon. mur | ʒ j. |
| | Tr. ferri mur | ʒ iv. |
| | Glycerini | ℥ iss. |
| | Syr. limonis | ℥ ij. |

M. Dose.—Teaspoonful.

If for a child under three years, reduce dose of all the remedies and dispense with the gargling, but give no liquids for ten or fifteen minutes after giving the liquid medicine.

This treatment will prove most satisfactory in every case, and comports well with the late theory that it is really a "germ disease," and *infectious*.

---

### TREATMENT OF DIARRHEA OF CHILDREN.

Livezey (quoted in *Mathews' Medical Quarterly*, January, 1894) states that in treating diarrhea of infants, children or adults, we should always remember that the secretions are defective, as indicated by a dry or coated tongue, unnatural color of stools, etc., and to attempt to arrest the watery discharges with such a pathological condition present by opiates and astringents will not last—will do injury, harm instead of good. Therefore first use:

℞ Hydrarg. chlorid. mitis............................gr. j.
Sodii bicarb.......................................gr. v.
Pulv. sacch. alb...................................gr. xx.
M. Ft. chart. x.

SIG.—One every two or three hours until discharges are changed in color and consistency; or hydrarg. cum cretâ, one part, triturated with two or three parts of sacch. lacti. Of this powder give two grains every two or three hours.

These powders will often restore healthy secretory action, and cure diarrhea alone. If not, follow with small doses of bismuth subcarb., nux vomica, and ipecac, or a few drops of the following:

℞ McMunn's elixir opii,

Tinct. rhei,

Tinct. camphoræ, } āā f ℥ ss.

M. SIG.—From five to ten drops every hour or two, as needed, and according to age of child.

Or, for very young children prescribe:

℞ Syr. rhei aromat...................................℥ j.

Tinct. opii camph...................................℥ ss.

Tinct. cardamom. comp...............................ʒ ij.

Aquæ calis..........................................℥ vj.

M. SIG.—Teaspoonful every hour or two, as needed.

—*College and Clinical Record.*

---

### THE USE OF BORIC ACID IN TYPHOID FEVER.

The *Union Médicale* gives a *résumé* of an article by Dr. L. Tortchinsky, published in the *Gaz. Hebdomadaire de Bordeaux* (New York *Medical Journal*):

The author used boric acid in two hundred and forty cases of typhoid fever in the course of an epidemic, and reports excellent results; only nine patients died, and they succumbed during the period of convalescence because they got out of bed too soon or committed errors in diet. The two hundred and thirty-one other patients made a rapid and complete recovery. In all the cases the patients were given a dose of castor oil with from five to ten drops of oil of turpentine. After this mixture had operated the administration of boric acid was begun, the remedy being given internally, either in powder or in solution, in doses ranging from twelve to fifteen grains for an adult three or four times a day. When there was bronchitis the boric acid was associated with expectorants and with hydrochloric acid. As a general rule, at the end of from three to five days the fever and the diarrhea underwent a noteworthy diminution, the tympanites disappeared, the dejecta lost their odor and became normal in appearance, the urine became abundant and in every way normal, the tongue and skin grew moist, and the general condition was good. As soon as the amelioration was well marked the use of the acid was discontinued and tonics were ordered. Under the influence of this treatment the disease followed a favorable course, its duration was somewhat diminished, and complications were very rare. The most decided effects were obtained in cases treated early. The author has found that the effect of the boric acid treatment may be increased by combining with that drug small doses of acetanilide, or salol. The mixture with quinine is especially useful in the last stages of the fever, when there are ataxia, delirium, and other cerebral symptoms; it is useful also in cases of relapse. The author has never observed any harmful effects from the use of boric acid. He has also produced satisfactory results with this acid in the treatment of the summer diarrhea of children.—*Medical Review.*

---

### SUDDEN DEATH FROM HEART-FAILURE.

The frequency of heart-failure as a cause of death has caused much comment from the laity, and brought forth a certain amount of grim humor concerning the diagnostic errancy of physicians. For it has been assumed that in most cases of sudden death doctors give heart-failure as a cause, in order to hide their ignorance of the true complaint. This view of the matter is unjust. Boards of Health require the statement of the immediate cause of death, and naturally in most acute maladies, as well as in some that are chronic, it is failure of the heart. But, aside from this, sudden death due directly and indirectly to failure of the heart is not very uncommon. In an article in the *Boston Medical and Surgical*

*Journal*, Dr. W. T. Councilman has gone over this question from the pathologist's point of view. Dr. Councilman does not consider cases of death due to the action of poisons or traumas; leaving out these, he enumerates the causes of sudden heart-failure somewhat as follows: First, emboli and thrombi developing in the heart or brought to it, and plugging its cavities; next, rupture of valves, or over-distention of the heart from too tight an aortic orifice; diseases of the coronary arteries, which lessen the lumen and cause degenerative changes in the muscle. As a result of this we have the so-called myocarditis, infarctions, and fatty degenerations. Rupture of the heart may occur from these last conditions, and rupture is not excessively rare as a cause of sudden death. Still, much the larger number of deaths in coronary disease is due to other causes than rupture of the muscle, and disease of the descending branch of the left coronary is most frequently present in these fatal cases.

Sudden death from heart-failure may be considered as most frequently due to coronary disease. Hence it is oftener seen somewhat late in life. Contrary to the usual belief, persons with valvular heart disease do not die suddenly, especially if it be the mitral valves that are affected.—*Medical Record.*

---

### PLEASANT LAXATIVES.

Every physician has felt the want of a pleasant purgative for children. Dr. H. C. Smith writes to the *Columbus Medical Journal* that the following formula has been used by him for years:

℞ Glycerine .......................................................... ʒ j.

Ol. cinnamon ..........................................................gtt. vj.

Trit. ben et adde.

Ol. rinci .......................................................... ℥i.

M. S. ʒ j at a dose p. r. n.

For chronic constipation Dr. Delafield prescribes the following pill:

℞ Strychniæ sulph..........................................................gr. 1-40.
Pulv. ipecac,
Ext. belladonna,
Ext. colocynth co. aa..........................................................gr. ¼.
M. Ft. pill No. i.
Sig. Four pills daily at first; then three, and afterwards two.

—*Brooklyn Medical Journal.*

### SOME PRESCRIPTIONS.

For urticaria:

℞ Liq. calcis.
Aq. lauro-cerasi
Glycerini, equal parts.

M. The skin is dabbed with some of the lotion, and then covered with a thin layer of cotton wool.—*Ex.*

---

For laryngeal phthysis:

| | | |
|---|---|---|
| ℞ | Iodoform | ʒ i. |
| | Pulv. acid. borici | gr. vii. |
| | Pulv. calcii phosph | ʒ iiss. |

M. Morning and evening the throat should be insufflated with the powder, which must be extremely fine.—*Ex.*

---

A mixture for asthma:

| | | |
|---|---|---|
| ℞ | Chloroformi | ʒ i. |
| | Etheris | ℥ iss. |
| | Syr. acaciæ | ʒ i. |
| | Tinct. cardamomi co. | ℥ i. |

M. A teaspoonful to be taken every half hour until relief is obtained.—*Ex.*

---

For tic douloureux:

| | | |
|---|---|---|
| ℞ | Ammonii chlordi | gr. xx. |
| | Butyl chloral hydratis | gr. v. |
| | Glycerini | m.x. |
| | Aquæ chloroformi, q. s., | ℥ ss. |
| | Misce et fiat haustus. | |

To be taken every two hours till three doses have been taken.—*Therap. Gazette.*

---

For fetid diarrhea in children:

| | | |
|---|---|---|
| ℞ | Calomel | gr. iss. |
| | Zinci sulpho-carbolatio | ʒ iiss. |
| | Bismuthi subnitratis | ℥ ii. |
| | Pepsinæ | gr. xxx. |

M. Divide into twenty powders; three powders daily for a child of one year.—*Ex.*

For nasal catarrh:

Professor Charles A. Wilson, of St. Louis, frequently uses the following, which can be varied to suit the case:

| | |
|---|---|
| ℞ Liq. alboline | ℥ ij. |
| Oil eucalyptus | gtt. x. |
| Terebene | ʒ ss. |
| Menthol | gr. v. |
| Oil gaultheria, q. s. to scent. | |

Use in compressed-air spray.—*Ex.*

---

Pills for chronic bronchitis:

| | |
|---|---|
| ℞ Ammonii chloridi | gr. xv. |
| Ammon. carb | gr. xv. |
| Pulv. ipecac | gr. iii. |
| Morph. hydrochloratis | gr. i. |
| Mucilaginis acaciæ | q. s. |

M. Divide into ten pills; one to be taken night and morning. —*Medical Press and Circular.*

---

A prescription for intermittent fever:

| | |
|---|---|
| ℞ Salicylate of quinine | gr. xx. |
| Syrup of orange | ℥ ii. |
| Rum | ℥ ii. |
| Simple syrup | ℥ iii. |

M. A dessertspoonful every hour for eight hours prior to the attack in quotidian fever, twelve hours prior in tertian fever, and fifteen hours before the attack in quartan fever.—*Journal de Médecine de Paris.*

